# Bioinformatics analysis of G protein subunit gamma transduction protein 2‐autophagy axis in CD11b+ dendritic cells as a potential regulator to skew airway neutrophilic inflammation in asthma endotypes

**DOI:** 10.1002/iid3.70038

**Published:** 2024-10-17

**Authors:** Xiaoying Ji, Yaoliang Zhou, Shendong He, Hongda Chen, Xianming Zhang, Zhifeng Chen, Jinwen Cai

**Affiliations:** ^1^ Department of Respiratory and Critical Care Medicine Affiliated Hospital of Guizhou Medical University Guiyang City Guizhou Province China; ^2^ Emergency and Disaster Medical Center The Seventh Affiliated Hospital, Sun Yat‐sen University Shenzhen City Guangdong Province China; ^3^ Department of Respiratory and Critical Care Medicine The First Affiliated Hospital of Guangxi University of Chinese Medicine, Xianhu District Nanning Guangxi China; ^4^ Department of Traditional Chinese Medicine The Seventh Affiliated Hospital, Sun Yat‐sen University Shenzhen City Guangdong Province China; ^5^ Department of Respiratory and Critical Care Medicine The Second Xiangya Hospital, Central South University Changsha City Hunan Province China; ^6^ Department of Respiratory and Critical Care Medicine The Third Xiangya Hospital of Central South University Changsha City Hunan Province China

**Keywords:** asthma phenotype, autophagy, dendritic cells, Gngt2, Th2, Th17

## Abstract

**Background:**

Asthma is a heterogeneous inflammatory disease with two main clinical endotypes: type 2 (T2) high and low asthma. The plasticity and autophagy in dendritic cells (DCs) influence T helper (Th)2 or Th17 differentiation to regulate asthma endotypes. Enhanced autophagy in DCs fosters Th2 differentiation in allergic environments, while reduced autophagy favors Th17 cell differentiation in sensitized and infected environments. Autophagy regulation in DCs involves interaction with various pathways like G protein‐coupled receptor (GPCR), mammalian target of rapamycin (mTOR), or phosphoinositide 3‐kinase (PI3K) pathway. However, specific molecules within DCs influencing asthma endotypes remain unclear.

**Methods:**

Gene expression data series (GSE) 64896, 6858, 2276, and 55247 were obtained from gene expression omnibus (GEO) database. Differentially expressed genes (DEGs) between CD103+ and CD11b+ DCs after induction by ovalbumin (OVA) and lipopolysaccharide (LPS) were analyzed using GEO2R. DEGs were examined through Gene Ontology (GO), Kyoto Encyclopedia of Genes and Genomes (KEGG), and protein–protein interaction (PPI) analyses. The hub gene network was construct with STRING database and Cytoscape. Autophagy differences in DCs and the selected hub gene in GSE6858, GSE2276, and GSE55247 were evaluated using student *t* tests.

**Results:**

Our analysis identified 635 upregulated and 360 downregulated genes in CD11b+ DCs, compared to CD103+ DCs. These DEGs were associated with “PI3K‐AKT signaling pathway,” “Ras signaling pathway,” and so forth. Thirty‐five hub genes were identified, in which G protein subunit gamma transduction protein 2 (Gngt2) in CD11b+ DCs exhibited a relatively specific increase in expression associated with autophagy defects under the induction environment similar to T2 low asthma model. No significant difference was found in lung Gngt2 expression between T2 high asthma model and control group.

**Conclusion:**

Our analysis suggested Gngt2 acted as an adapter molecule that inhibited autophagy, promoting Th17‐mediated airway inflammation via the GPCR pathway in a T2 low asthma mice model. Targeting this pathway provides new asthma treatment strategies in preclinical research.

## Graphical abstract

We illustrate a novel mechanism involving Gngt2 in DC‐mediated autophagy‐axis in T2‐low asthma.

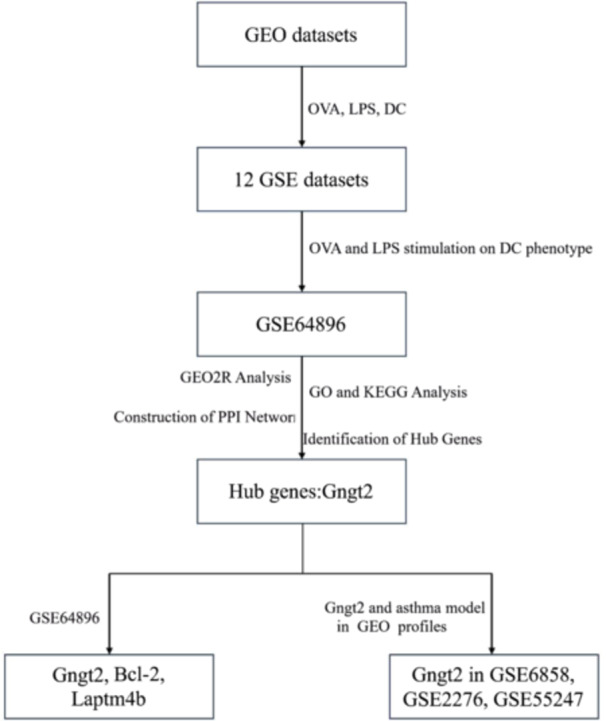

## 1 INTRODUCTION

Asthma, characterized by airway inflammation involving various cells like DCs, CD4+ Th cells, and granulocytes, is traditionally categorized into eosinophilic, neutrophilic, mixed granulocytic, and paucigranulocytic phenotypes.^1^, ^2^, ^3^ Notably, airway neutrophilic inflammation is commonly associated with severe asthma and limited response to glucocorticoid therapy.^4^, ^5^ Tailoring treatments to specific asthma phenotypes can improve asthma control and reduce exacerbations.^6^ Therefore, a deeper understanding of the regulatory mechanisms underlying these asthma inflammation phenotypes is essential.

Recently, attention has shifted from inflammatory phenotypes to complex biological networks linked to T2 high or low endotypes.^7^ T2‐high asthma, the most prevalent type, involves a Th2‐driven immune response characterized by cytokines interleukin(IL)−4, 5 and 13 along with elevated serum IgE, blood eosinophilia, and eosinophilic lung inflammation induced by allergens like OVA or house dust mites (HDM) in mouse models.^3^, ^8^ Inhibiting IL‐4 or IL‐13 pathways significantly reduces airway eosinophilic inflammation in asthmatic mice.^9^ Conversely, T2‐low asthma lacks typical T2 characteristics, showing neutrophils or normal eosinophil levels in sputum, biopsies, or blood samples.^10^ It comprises a substantial portion of asthma cases and correlates significantly with airway neutrophilic inflammation driven by LPS levels or airway bacterial burden.^11^, ^12^, ^13^ Combining OVA with LPS induces a shift from eosinophilic to neutrophilic inflammation in asthma models, mediated by Th17 cells and cytokines like IL‐17, IL‐21, and IL‐22.^10^, ^14^, ^15^

Compared to T2‐low asthma, patients with T2 high asthma tend to be younger (OR 0.945), have a lower body mass index (OR 0.913), lower smoke exposure (OR 0.975), and a higher incidence of rhinitis (OR 3.491) (*p *< .001).^16^ Furthermore, T2‐low asthma is associated with clinical features such as later onset, obesity, glucocorticoid resistance, and increased exacerbation risk.^10^ Glucocorticoid therapy effectively targets Th2‐driven eosinophilic inflammation but has limited impact on Th17‐driven neutrophilic inflammation.^17^ Therefore, T2‐high asthma is easily induced by allergic stimuli, while T2‐low asthma is specifically associated with allergic and infectious environments. Understanding Th2/Th17 differentiation mechanisms is crucial for insight into eosinophil and neutrophil recruitment and transformation in T2‐high or low asthma.

Recent studies highlight the crucial role of DCs in regulating asthma‐related immune responses. DCs in the lung are classified as classical DCs (cDCs), comprising CD103+/CD11b+ DCs subgroups, and plasma cell‐like DCs (pDCs). Environmental triggers can alter DC subgroup proportions. For instance, inhaled allergens increase bone marrow DC precursor cells and their migration to the airway, resulting in elevated CD103+ and CD11b+ DCs in bronchoalveolar lavage fluid, whereas only CD11b+ DCs increase in the lung.^18^ Under LPS stimulation, CD103+ DCs can transition into CD11b+ DCs^19^ and peripheral monocytes can migrate to the lungs and differentiate into CD11b+ DCs.^20^ Functionally, DC phenotypes play distinct roles in regulating Th cell differentiation.^21^ Antigens fail to induce Th2 differentiation in the absence of CD103+ DCs.^22^, ^23^ In asthma mouse models, HDM binding to the surface receptor Dectin on CD11b+ DCs promotes Th2/17 inflammation. Conversely, in mice lacking the Dectin surface receptor on CD11b+ DCs, there's a reduction in lung Th2/17 cells and diminished concentrations of interleukin‐5, 13, and 17 in alveolar lavage fluid.^24^, ^25^ In asthma models with OVA/diesel exhaust particles (DEPs) or HDM/DEP co‐exposure, neutrophilic airway inflammation was accompanied by increased percentages of CD11b+ dendritic cells (DCs) in the lung tissue.^26^, ^27^ In OVA‐exposed female asthma mice models, enhanced Th2 cytokine production may be attributed to CD103+ DCs in the bronchial lymph nodes.^23^ These studies identify the potential roles of CD11b+ DCs in mucosal Th17 immune responses and CD103+ DCs are crucial for inducing airway Th2 inflammation in asthmatic mice.^28^, ^29^

Autophagy dysregulation in DCs significantly impacts immune regulation in asthma, Stimuli like OVA enhance autophagy‐related proteins (e.g., autophagy‐related proteins‐5 (ATG5), light chain(LC)3‐II, promoting Th2 responses.^30^ while HDM triggers Th17 differentiation via the IL‐1 and IL‐23 pathways in ATG5‐deficient DCs.^21^ Conversely, LPS stimulation, an asthma adjuvant in airway immunization, inhibited DC autophagy and promoted Th17 cell differentiation.^31^, ^32^ On the other hand, genetic polymorphisms in ATG5 (−769T>C, −335G>A, and 8830 C>T) and ATG7 (−100A>G and 25108 G>C), were correlate with neutrophilic airway inflammation in adult asthma.^33^ Balancing autophagy in DCs may modulate asthma endotypes by regulating Th cell differentiation.

Based on the above studies mentioned, a critical inquiry emerges: how to find the key autophagy related molecule in DCs that modulates the status of autophagy and influences Th cell differentiation in T2 low asthma model?

## 2 MATERIALS AND METHODS

### 2.1 Data preprocessing

Gene expression omnibus is a high‐throughput gene expression database. To investigate the role of DCs phenotype in T2 low asthma, we searched in the GEO data sets using OVA, LPS, DC as keywords and obtained 12 data sets. Among the retrieved data sets, we only found that GSE64896 explored the effects of OVA and LPS stimulation on DC phenotype.

### 2.2 Identification of differentially expressed genes

For this study, we retrieved and analyzed gene expression data from the GSE64896 data set, employing GEO2R to discern DEGs between CD103+ and CD11b+DCs by OVA and LPS induction. We applied stringent criteria, setting an adjusted *p* < 0.01 and |log2 (fold change)| > 1 to identify significant DEGs. Visualization of DEGs analysis was achieved through volcano plots.

### 2.3 Gene ontology and kyoto encyclopedia of genes analysis

The GO analysis categorizes and describes gene functions across biological processes (BP), cellular components (CC), and molecular functions (MF). Meanwhile, KEGG analysis uses a comprehensive database that integrates genomic, chemical, and systemic functional information to identify significant biological pathways associated with differentially expressed genes. In this study, we utilized R software (version 3.6.2) along with several packages including clusterProfiler, enrichplot, ggplot2, and org.Mm.eg.db to conduct GO and KEGG analyses. The resulting bubble chart visually represents the significance of primary signaling pathways in biological functions, depicted by circle size and adjusted *p* values. Statistical significance was determined at an adjusted *p* < .05.

### 2.4 Construction of protein–protein interaction network and identification of hub genes

To explore potential interactions among the DEGs, we constructed a PPI network utilizing the STRING database (https://string-db.org/, downloaded on January 10, 2023). In this analysis, we specified “Mus musculus” as the organism and set a minimum required interaction score of ≥0.9 as the criterion for screening interactions. The resultant PPI network offered insights into the connections among DEGs. To pinpoint key genes in asthma endotype, we conducted topology analysis of the PPI network using the CytoNCA plugin within Cytoscape software. Hub genes were determined based on degree centrality.

### 2.5 The comparison of autophagy related genes and Hub gene expression in asthma

**Figure 1:**
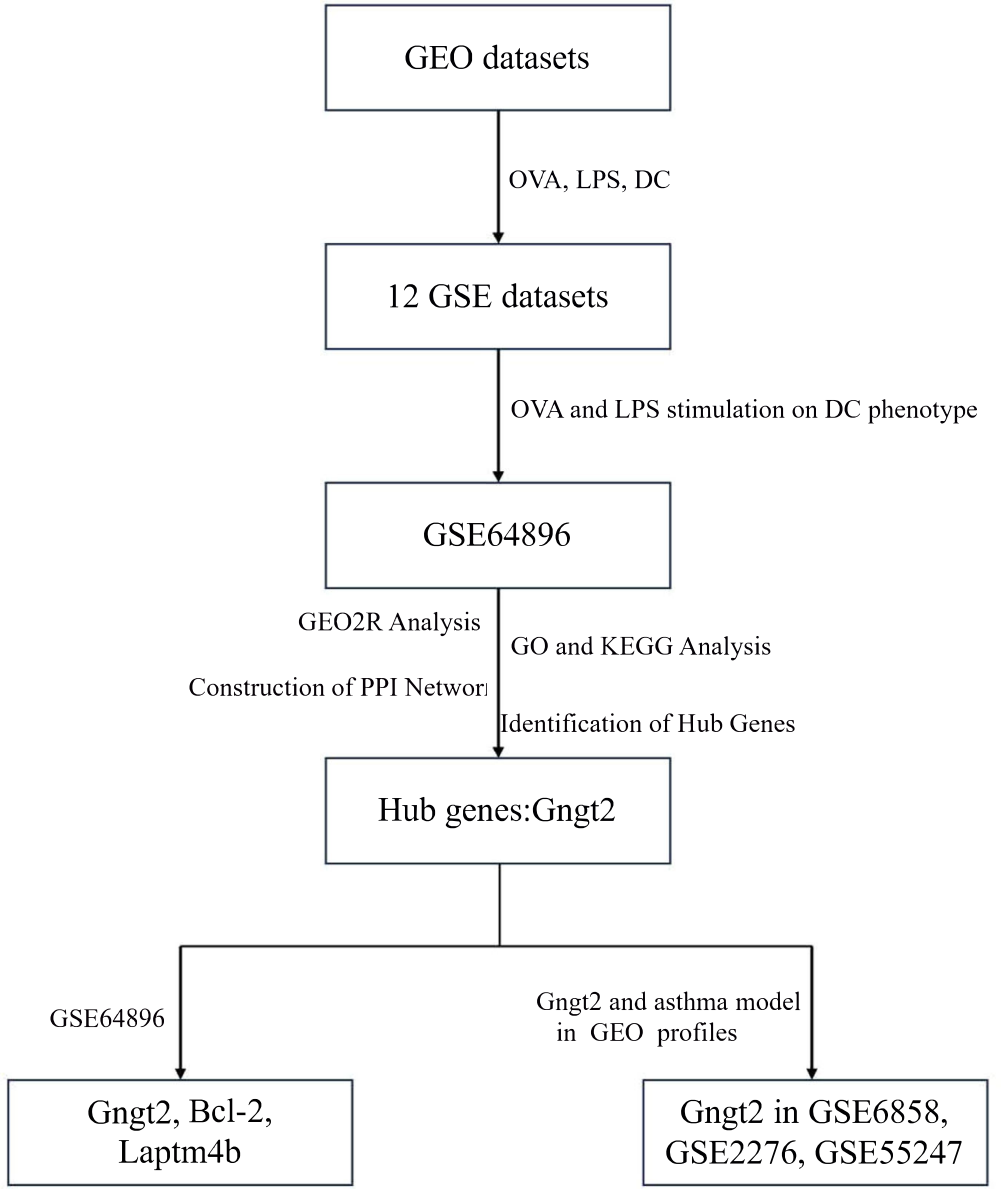
The entire workflow of the research method.

To assess the relationship between autophagy related genes and Hub gene, the expression variances of Gngt2, B‐cell leukemia/lymphoma‐2(Bcl‐2), and lysosomal‐associated protein transmembrane 4B (Laptm4b) in the GSE64896, were individually compared using two‐sided Student *t* tests. Additionally, to further investigate the expression of the selected Hub gene in T2 high asthma model, we searched in the GEO profiles using the selected Hub gene and asthma model as keywords and found GSE6858, GSE2276, GSE55247 data sets. In the three datasets, the expression of Gngt2 in the lung between control group and OVA induced asthma model were individually compared using two‐sided Student *t* tests. The entire workflow of the research method is shown in (Figure 1).

### 2.6 Statistical analysis

We conducted statistical analysis utilizing two‐sided Student's *t* tests, and the data were presented as means ± standard deviation. A two‐sided *p* < .05 was regarded as statistically significant.

## 3 RESULTS

### 3.1 Identification of hub genes in OVA and LPS‐stimulated CD11b+ and CD103+ DCs

**Figure 2:**
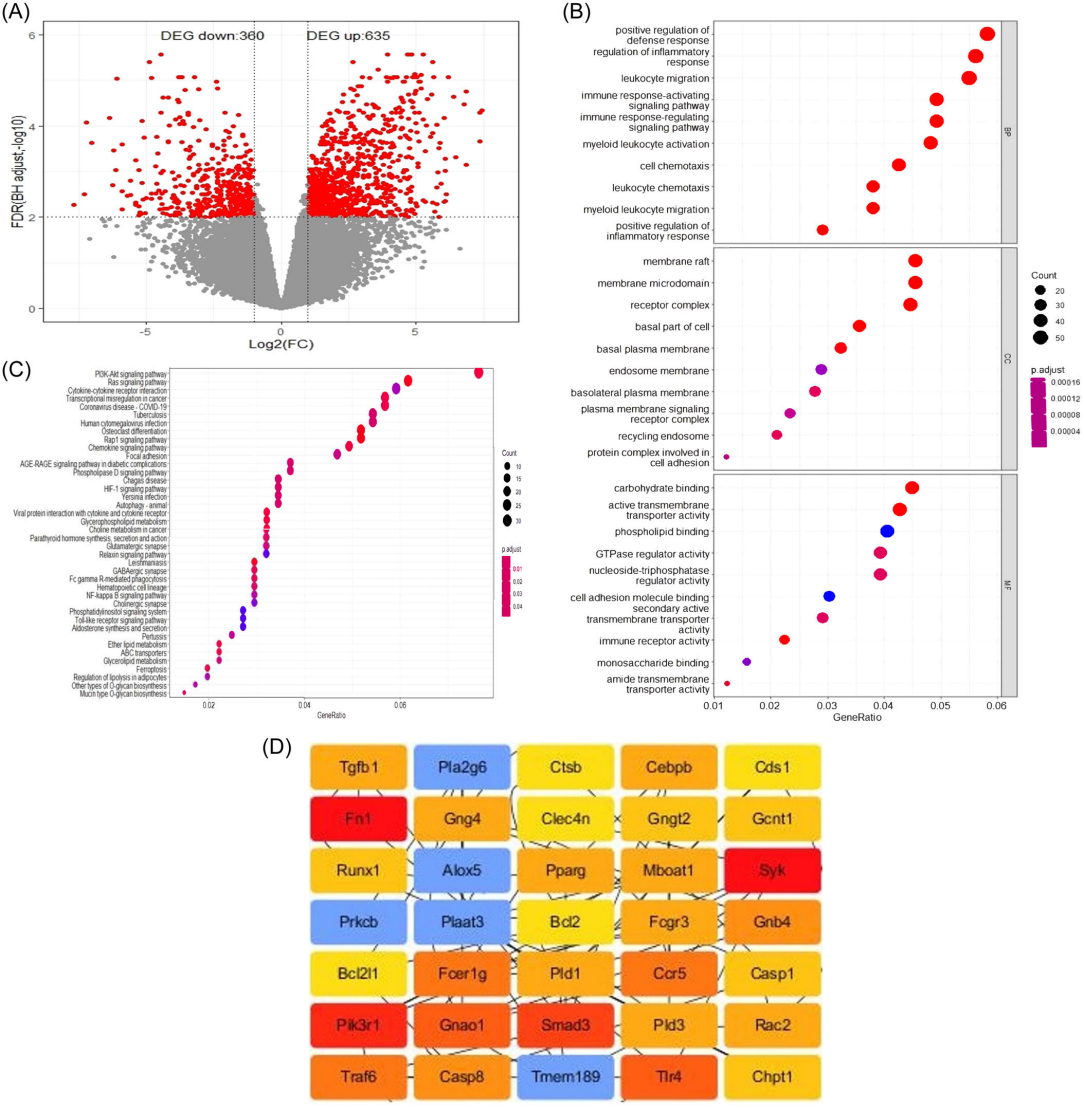
Differentially expressed genes analysis and Hub genes identification between CD103+ and CD11b+ dendritic cells (DCs) stimulated by ovalbumin and lipopolysaccharide in Gene Expression data Series 64896. (A) Volcano plot of 995 differentially expressed genes in Gene Expression data Series 64896. (B) Gene ontology analysis of 995 differentially expressed genes. (C) Kyoto encyclopedia of genes and genomes analysis of 995 differentially expressed genes. (D) Network of 35 Hub genes in gene expression data series 64896.

In our study, we conducted a DEGs analysis from the GSE64896 data set, focusing on DCs stimulated by OVA and LPS. We identified 995 DEGs, comprising 635 upregulated and 360 downregulated genes (Figure 2A and Supporting Information S1: Material 1).

To elucidate the biological significance of these DEGs, we performed enrichment analyses to annotate their BP, CC, and MF. The top six BP enrichments included responses related to “positive regulation of defense response,” “regulation of inflammation response,” “leukocyte migration,” “immune response‐activating and regulating signaling pathways,” and “myeloid leukocyte migration” (Figure 2B). The top six CC enrichments comprised responses associated with “membrane raft,” “membrane microdomain,” “receptor complex,” “basal part of cell,” “basal plasma membrane,” and “endosome membrane” (Figure 2B). The top six MF enrichments involved responses related to “carbohydrate binding,” “active transmembrane transporter activity,” “phospholipid binding,” “GTPase regulator activity,” and “nucleoside‐triphosphatase regulator activity” (Figure 2B).

Additionally, we conducted KEGG pathway analysis to uncover pathways associated with these DEGs. The top six enriched pathways included the “PI3K‐AKT signaling pathway,” “Ras signaling pathway,” “cytokine‐cytokine receptor interaction,” “transcriptional misregulation in cancer,” “Coronavirus disease‐COVID‐19,” and “Tuberculosis” (Figure 2C). To explore interactions among these DEGs, we constructed a PPI network using the STRING database and Cytoscape software. The PPI network, displayed in Supporting Information S2: Material 2, identified 35 hub genes based on their Degree means (Figure 2D).

### 3.2 Expression of Gngt2 and autophagy‐related genes in asthma

**Figure 3:**
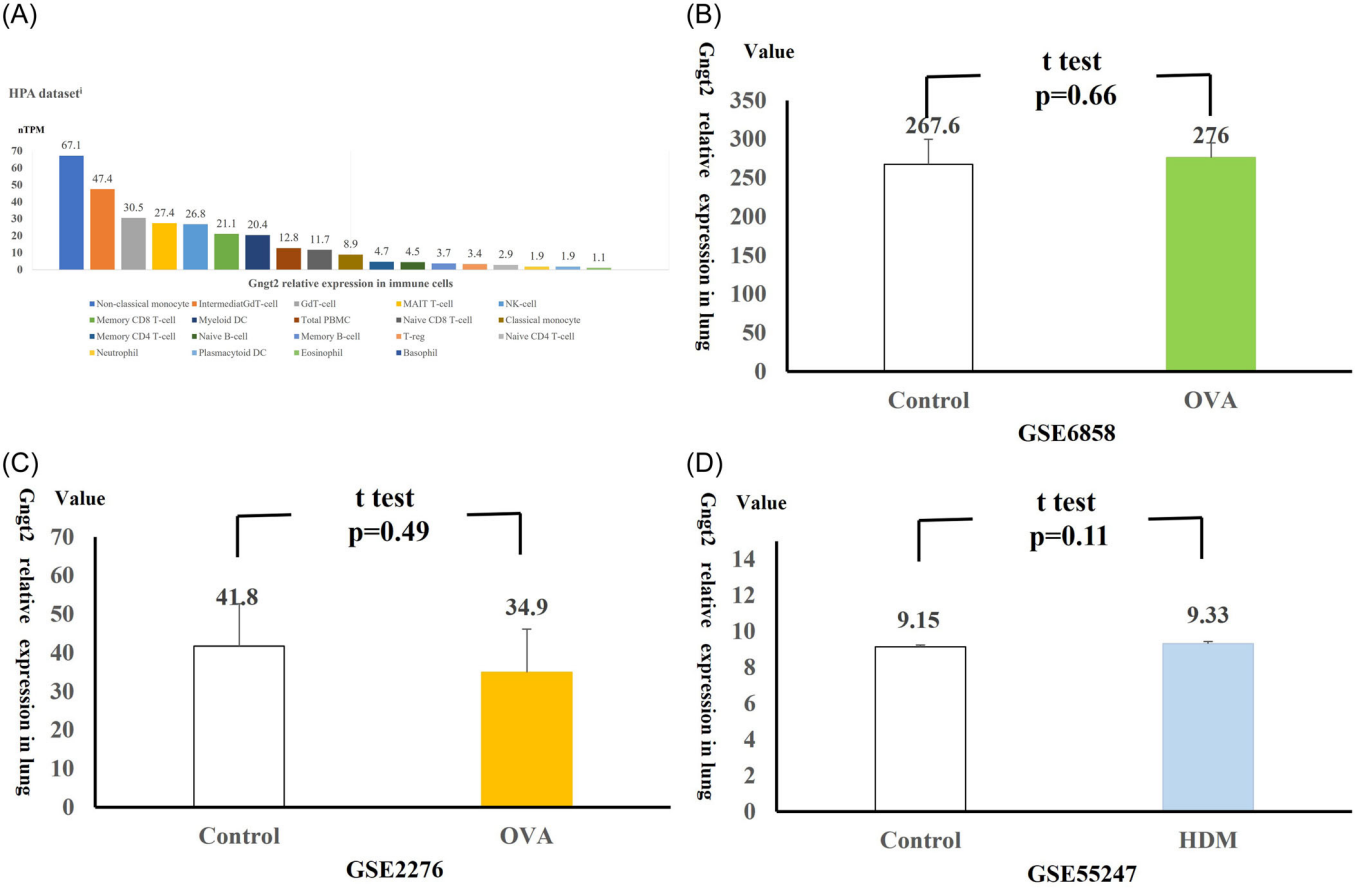
G protein subunit gamma transduction protein 2 (Gngt2) expression in different data sets. (A) Gngt2 expression levels of different immune cells in the human protein atlas (HPA）database. (B) Lung Gngt2 expression levels in gene expression data series 6858. (C) Lung Gngt2 expression levels in gene expression data series 2276. (D) Lung Gngt2 expression levels in gene expression data series 55247.

Among these hub genes, Gngt2 was notable for its role within GPCR family, specifically as a G‐protein γ subunit located on human chromosome 17q21.^34^ It likely served as an adaptor protein, facilitating upstream signal transduction to downstream elements in the GPCR pathway. The human protein atlas (HPA) database highlighted Gngt2's immune cell specificity, showing high enrichment in nonclassical monocytes and moderate expression in myeloid DCs (Figure 3A). To better understand Gngt2 expression in asthma model, we conducted further analysis on lung Gngt2 expression using the GSE6858, GSE2276, GSE55247 data sets. All asthma model in datasets were T2 high asthma model induced by OVA or HDM. Our analysis revealed no significant difference in lung Gngt2 expression between the OVA or HDM‐stimulated group and the control group (GSE6858 OVA: 276 ± 19, control: 267.6 ± 32, *p *= .66; GSE2276 OVA: 34.9 ± 11.2, control: 41.8 ± 10.9, *p *= .49, GSE55247: HDM: 9.33 ± 0.10, control: 9.15 ± 0.10, *p *= .11) (Figure 3B–D). These findings suggested that Gngt2 may play a regulatory role in allergen and infection induced CD11b+ dendritic cells.

**Figure 4:**
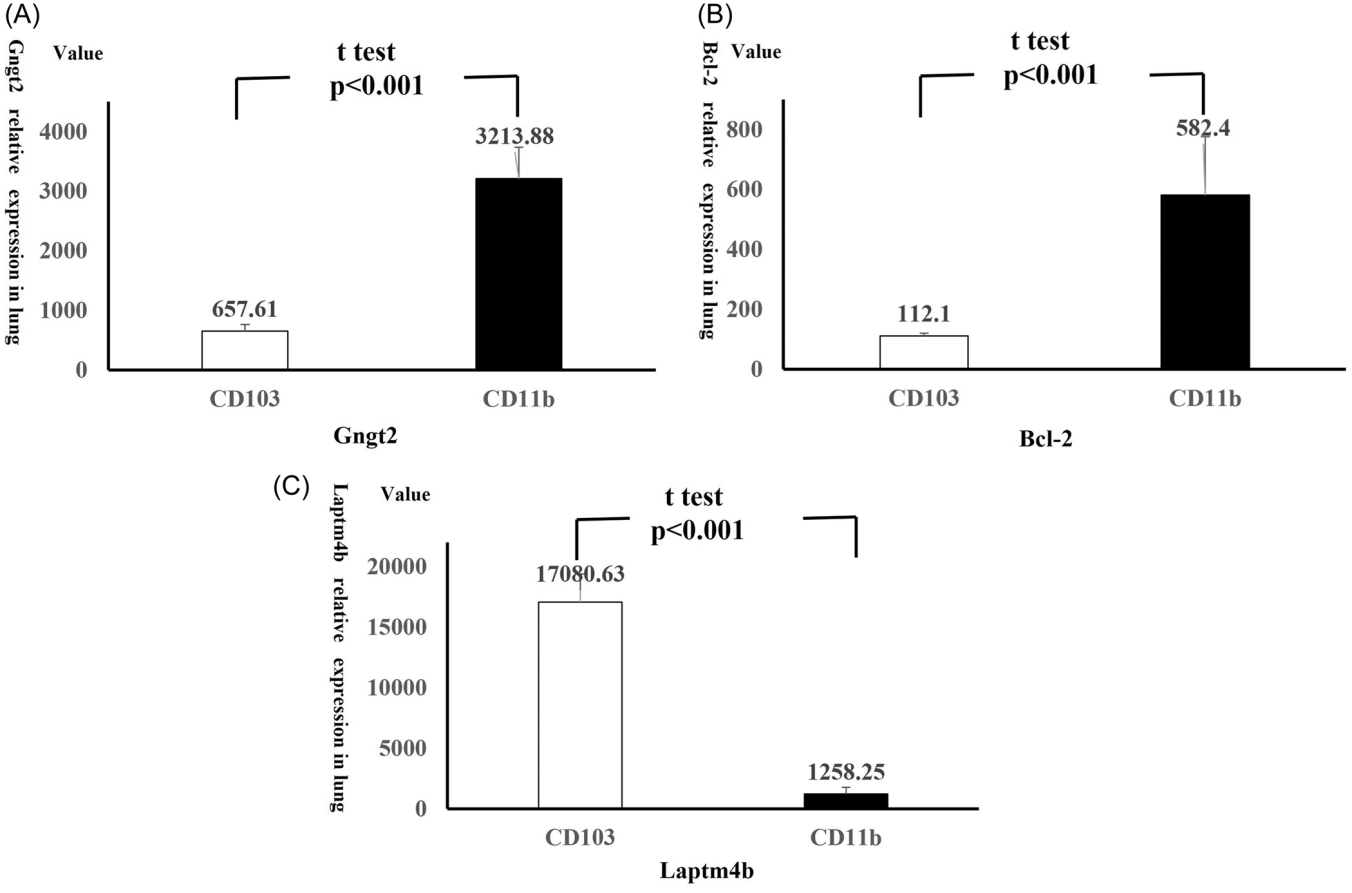
G protein subunit gamma transduction protein 2 (Gngt2), B‐cell lymphoma‐2(Bcl‐2), and lysosomal‐associated protein transmembrane 4B (Laptm4b) expression between CD103+ and CD11b+ dendritic cells (DCs) stimulated by ovalbumin and lipopolysaccharide in Gene Expression data Series 64896. (A) Gngt2 expression levels between CD103+ and CD11b+ dendritic cells in gene expression data series 64896. (B) Bcl‐2 expression levels between CD103+ and CD11b+ dendritic cells in gene expression data series 64896. (C) Laptm4b expression levels between CD103+ and CD11b+ dendritic cells in gene expression data series 64896.

A previous study has indicated that Gngt2 deficiency can promote cell proliferation in tumor cells by upregulating Bcl‐2 expression.^35^ This suggested a potential role for Gngt2 in regulating autophagy through its interaction with Beclin1 and Bcl‐2. Our bioinformatics analysis also revealed a correlation between Gngt2 expression and changes in autophagy‐related genes. Specifically, Gngt2 expression was significantly elevated in CD11b+ DCs following OVA and LPS stimulation compared to CD103+ DCs (CD103: 657.61 ± 109.46, CD11b: 3213.88 ± 527.94, *p *< .0001) (Figure 4A). Furthermore, Bcl‐2 expression showed a significant increase (CD103: 112.10 ± 8.32, CD11b: 582.40 ± 192.98, *p *< .0001) (Figure 4B), while Laptm4b expression was significantly reduced (CD103: 17080.63 ± 2342.68, CD11b: 1258.25 ± 361.95, *p *< .0001) (Figure 4C) in CD11b+ DCs. These findings suggest that Gngt2 may interact with impaired autophagy and lysosomal activity in CD11b+ DCs.

## 4 DISCUSSION

DCs are widely recognized as specialized antigen‐presenting cells that establish the microenvironment conducive to CD4+ T‐cell differentiation. This is achieved through the recognition and capture of antigens by pathogen recognition receptors, followed by antigen processing via autophagy. This process involves the production of inflammatory cytokines and the upregulation of costimulatory molecules. A microenvironment that supports Th2 differentiation is characterized by low‐dose antigen presentation, reduced levels of IL‐12, and a cytokine milieu rich in IL‐4. The expression of co‐stimulatory molecules such as OX‐40L, Jagged1, and CD40 on DC surfaces is implicated in this process. Cytokines like IL‐25, IL‐33, and thymic stromal lymphopoietin can upregulate the expression of OX‐40L and Jagged1 in DCs, thereby promoting Th2 differentiation.^36^, ^37^, ^38^ Conversely, Th17 differentiation is facilitated by high‐dose antigen presentation, the presence of co‐stimulatory factors such as CD40 and CD86 on DC surfaces, and a cytokine‐rich environment comprising transforming growth factor (TGF)‐β, IL‐1, IL‐6, and IL‐23.^39^ Additionally, the plasticity of DC sub sets allows them to adapt their functions to different asthma endotypes when exposed to allergens or a combination of allergens and bacterial burdens. CD11b+ DCs express higher levels and a broader range of chemokines, while CD103+ DCs specialize primarily in capturing inhaled antigens and facilitating the recruitment and proliferation of CD4+ T cells in the draining lymph nodes of the airways, particularly during allergic airway inflammation episodes.^40^, ^41^, ^42^, ^43^

Sensu stricto autophagy, also referred to as macroautophagy, represents a multifaceted cellular process characterized by heightened levels of endocytosis and lysosomal degradative activity to maintain organelle and protein homeostasis.^44^ Notably, autophagic activity in DCs plays a critical role in the differentiation and activation of Th cells, as indicated in Supporting Information S3: Material 3. Interestingly, the balance of autophagy in DCs influences the type of airway inflammation. OVA‐induced DCs autophagy occurred in a dose‐dependent manner, promoting a Th2‐driven immune response.^45^ Respiratory syncytial virus (RSV) infection has been associated with an increased risk of developing allergic asthma and T2 high inflammation.^46^ However, RSV‐infected LC3b^−/−^ DCs exhibited downregulation in autophagosome formation, leading to increased IL‐17 secretion when co‐cultured with CD4+ T cells compared to wild‐type DCs.^47^ Therefore, augmented autophagy in DCs promotes Th2 differentiation within a sensitized microenvironment, whereas diminished autophagy within DCs is more inclined to foster Th17 cell differentiation, particularly in a sensitized environment that is concurrently infected with bacteria.^21^

The induction of autophagy can be triggered by environmental or stress‐related stimuli including energy status, hypoxia, oxidative stress, pathogens, and mechanical or growth factor challenges. During cellular starvation, basal autophagy undergoes robust induction by inhibiting mTOR in response to catabolic signals. Conversely, in the presence of sufficient nutrients, autophagy is curtailed to facilitate protein synthesis by reactivation of mTOR.^48^ Pathogens modulate a cascade of signaling pathways, including mitogen‐activated protein kinase (MAPK) or PI3K, ultimately culminating in mTOR inhibition and autophagy promotion.^49^, ^50^ Decreased intracellular ATP levels activate adenosine 5'‐monophosphate (AMP)‐activated protein kinase (AMPK), a sensor of adenosine nucleotide levels, which induces autophagy either by inhibiting mTOR or directly activating autophagic processes.^51^ Upon sensing nutrient signals, stimulation of the Gαq/11‐coupled GPCR pathway activates mTOR to counteract autophagy induced by serum or amino acid deprivation.^52^ Following the regulation of these signaling pathways, autophagy entails the activation of multiple ATG and kinase interactions to initiate internal membrane formation, including phagophore initiation, phagophore elongation, autophagosome formation, and autophagolysosomal fusion.^53^ For example, Bcl‐2 was a well‐established regulator of autophagy, to form inhibitory complexes with Beclin 1.^54^ The upregulation of Bcl‐2 is associated with the secretion of Th17‐inducing factors like IL‐6 or IL‐23, often accompanying autophagy deficiency.^55^, ^56^ The downregulation of LAPTM4B contributed to impairment of autophagic flux via modulation of the mTOR pathway.^57^ In our study, we found increased Bcl‐2 expression and Laptm4b reduced expression in CD11b+ DCs, compared with CD103+ DCs, which indicated impaired autophagy existed in CD11b+ DCs. Therefore, mTOR serves as a central regulatory point in autophagy, interconnected with other signaling pathways like GPCR. The autophagy related proteins like ATG, Bcl‐2, Beclin 1 and so forth. serve as markers for alterations in autophagic activity and regulating Th17 differentiation by affecting the production of inflammatory cytokines.

Previous research has highlighted the pivotal role of GPCR pathways in regulating asthma pathophysiology through their influence on various factors such as airway smooth muscle tone and immune cell function. Central to these pathways is the G protein, comprising three subunits: G_α_, G_β_, and G_γ_. In the inactive state, these subunits form a stable heterodimer with GDP. When a GPCR is activated by an external stimulus, it sequentially activates heterotrimeric G proteins to trigger conformational changes. The G_α_ subunit binds to GTP and activates downstream effector proteins after releasing GDP. Simultaneously, the G_β_ and G_γ_ subunits dissociate from the heterotrimeric G protein. The signaling process terminates when the G_α_ subunit hydrolyzes GTP to GDP, a reaction catalyzed by regulator of G protein signaling (RGS) proteins. After hydrolysis, the G_α_ and G_βγ_ subunits reassociate, reforming the G_αβγ_ heterotrimer.

There are four major classes of specific Gα subunits (G_i/o_, G_s_, G_q/11_, G_12/13_) acting as molecular switches that determine the activation of downstream signaling cascades within the cell.^58^ For example, G_αs_ activates adenylyl cyclase (AC) thereby elevating cyclic adenosine monophosphate (cAMP) levels and activating protein kinase A (PKA). Conversely, G_αi_ inhibits AC, leading to decreased cAMP levels. G_αq/11_ stimulates the generation of diacylglycerol (DAG) and inositol trisphosphate, resulting in the release of Ca^2+^ from the endoplasmic reticulum through the activation of phospholipase Cβ (PLCβ). Apart from the two effectors, the Gαq/11 subfamily also includes noncanonical effectors such as protein kinase Cζ and p63 RAS homology (Rho) guanine nucleotide exchange factor (p63RhoGEF).^59^, ^60^ Finally, Gα12/13 were found to directly interact with guanine nucleotide exchange factors (GEFs) and activate small GTPases, including the small GTP‐binding protein Rho and Rho kinas.^61^ During GPCR signal transduction, a single GPCR can couple to several G proteins, allowing it to activate multiple downstream signaling pathways. Therefore, inhibition of the GPCR pathway can block a series of downstream signaling pathways. On the other hand, an activated GPCR can couple not only to G proteins but also to other effectors like PI3K or mTOR, resulting in regulating additional signaling pathways.^59^ This phenomenon, known as biased agonism or functional selectivity, has been observed in different types of GPCRs, which may be closely associated with asthma endotypes.^62^

Previous studies have implicated dysfunction of GPCRs in asthmatic features such as excessive contraction in airway smooth muscle (ASM), hyperplastic/hypertrophic ASM and airway inflammation.^63^ In addition to regulate ASM, GPCR pathways also play a role in immunomodulatory functions in asthma. For instance, leukotrienes, including cysteinyl leukotrienes (CysLTs) and LTB_4_, could stimulate CysLTs receptors (CysLTRs) to mediate neutrophil chemotaxis or activate eosinophils in the asthmatic airway through specific interaction with G_i/o_ or G_αq/11_.^64^, ^65^ Importantly, GPCR pathways can directly or indirectly regulate Th cell activation and differentiation by multiphase cross‐regulating pathways.^66^, ^67^ In DCs, G_αs_ and G_αi_ activation regulate the induction and regulation of Th2 immunity and allergic asthma by modulating cAMP balance. The deletion of G_αs_ decrease cAMP, provoke Th2 polarization and yields a prominent allergic phenotype.^66^ Additionally, Gαq subunit deficiency in DCs significantly reduces IL‐6 production and inhibits Th17 differentiation.^67^ The deletion of CysLTRs in DCs results in reduced production of pro‐inflammatory cytokines, including IL‐6, TNF‐α, and IL‐12, and decreased Th1 and Th17 cell differentiation through LTB_4_‐CysLTRs‐ G_αi_‐PKC pathway.^68^

**Figure 5:**
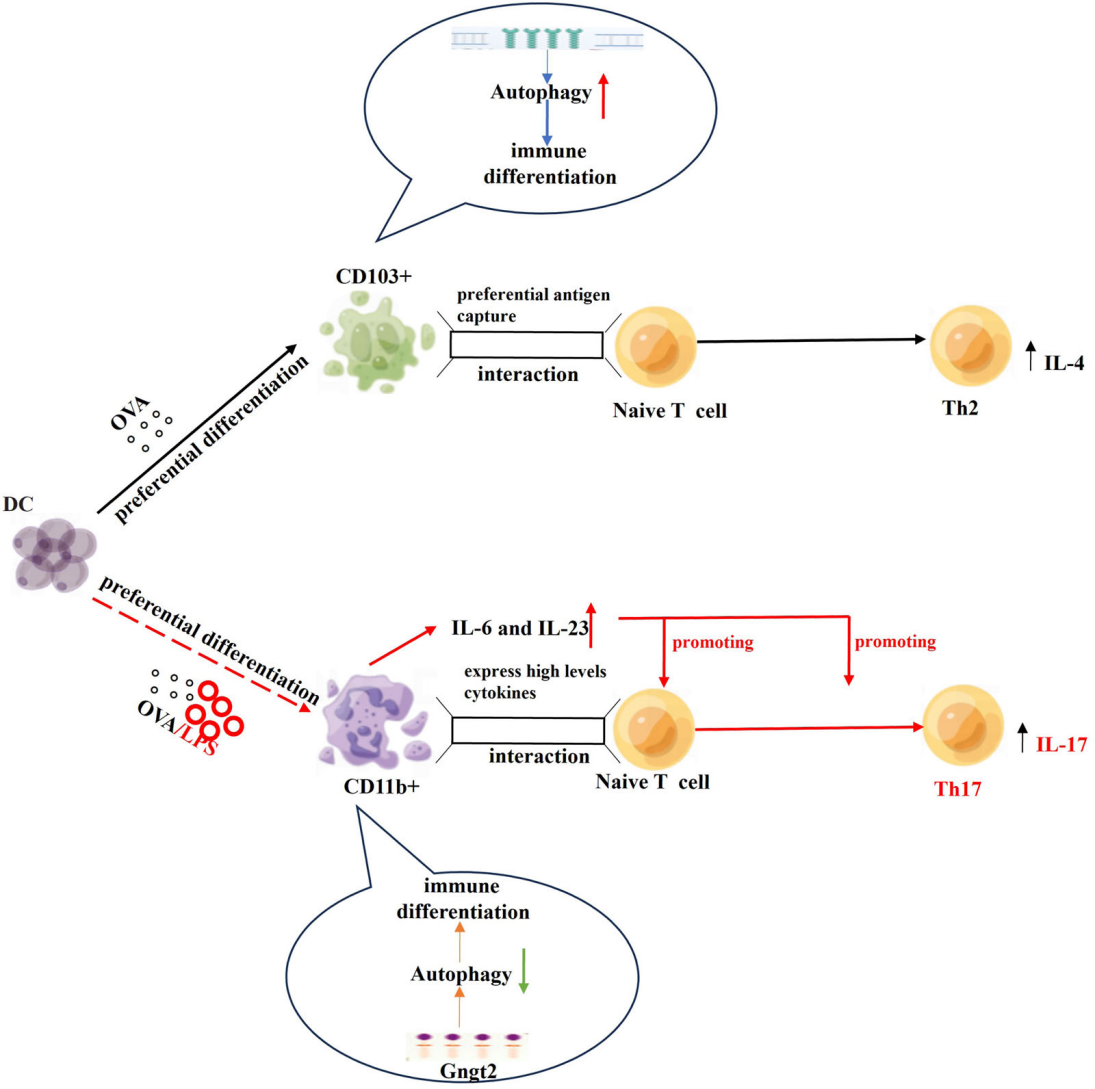
The graphical abstract of G protein subunit gamma Transduction protein 2 (Gngt2)‐autophagy Axis in CD11b+ dendritic cells (DCs) regulating asthma endotypes. In T2 high asthma, dendritic cells differentiate toward CD103+ types under allergens like ovalbumin, promoting autophagy and Th2‐driven airway inflammation. In T2 low asthma, dendritic cells turn into CD11b+ types under allergens and infections, inhibiting autophagy and producing IL‐6 and IL‐23 via G protein‐coupled receptors, which drive Th17‐mediated inflammation.

Compared to Gα proteins, Gγ protein and its related subunits in DCs are less studied. The specific mechanism involving Gngt2 remains uncertain, although some reports suggest that Gγ subunits interact with various binding partners to influence the GPCR pathway.^69^ Several studies have identified Gngt2 as a risk gene associated with moderate to severe asthma, but its role in severe asthma is unclear.^70^, ^71^ The above‐mentioned studies and our bioinformatics analysis indicate that Gngt2 is closely relation with GRCP pathways and autophagy. Considering the distinct roles of DC phenotypes and autophagy balance in Th differentiation, we propose a possible regulatory mechanisms: in T2 high asthma, DCs differentiate towards CD103+ DCs under long‐term allergen induction such as OVA, while in T2 low asthma, DCs are more likely to differentiate towards CD11b+ DCs under allergen and infection background. The predominant expression of gngt2 in CD11+ DC cells function as an adapter molecule to inhibit autophagy for its interaction with Bcl‐2 by GPCR pathway, thereby promoting Th17‐mediated airway neutrophilic inflammation in T2 low asthma(Figure 5).

## 5 SUMMARY, OUTLOOK, AND CONCLUSION

In asthma, GPCRs pathways are involved in the whole respiratory tract to regulate both ASM contraction and airway inflammation. Ideally, drugs targeting GPCRs in asthma should mitigate airway inflammation and prevent excessive ASM contraction, yet no such drug currently exists. The current GPCR‐related drugs for asthma treatment, such as β‐agonists, muscarinic antagonists, leukotriene modifiers, and theophylline, primarily target individual GPCRs, posing a limitation. Combination therapy may be necessary for severe asthma cases, and existing drugs predominantly address T2‐high asthma inflammation, neglecting T2‐low asthma inflammation. However, our research suggests that controlling T2‐low inflammation through GPCRs is feasible, as multiple GPCRs contribute to DC‐Th17‐neutrophil inflammation. Thus, there's an urgent need for novel therapeutic strategies to address adverse reactions and suboptimal disease control in asthma patients.

In our study, we illustrate a potential mechanism involving Gngt2 in DC‐mediated autophagy‐axis as a potential therapeutic intervention for T2‐low asthma. Although there is supportive evidence for our hypothesis, further experiments are needed for its validation. We outline four main parts of our experimental approach. First, we observe the changes in Gngt2 and downstream autophagy in DCs using OVA‐induced T2‐high and OVA‐LPS‐induced T2‐low asthma models. Second, we verify the effects of Gngt2 intervention on autophagy and asthma endotypes. Subsequently, we demonstrate that Gngt2 in DCs can regulate Th2/17 differentiation through the autophagy pathway. Finally, we confirm that the modulatory effects of Gngt2 signaling on Th2/17 differentiation in asthma endotypes depend on autophagy.

To date, the role of GPCR pathways in asthma endotype pathogenesis remains understudied, which need more experiments to verify. The attractive properties of GPCR ligands as therapy targets offer an alternative to corticosteroid treatment for asthma, potentially minimizing associated side effects.

## AUTHOR CONTRIBUTIONS

Xiaoying Ji contributed to manuscript writing and data analysis. Yaoliang Zhou contributed to drawing pictures. Shendong He interpreted and analyzed the data, while Hongda Chen critically reviewed the manuscript. Xianming Zhang, Zhifeng Chen, and Jinwen Cai designed the study and provided critical review. All authors participated in the literature search and collaborated on developing the theory presented in the manuscript.

## CONFLICT OF INTEREST STATEMENT

The authors declare no conflict of interest.

## ETHICS STATEMENT

All authors provided their consent to publish our article in *Journal of Immunity, Inflammation and Disease*.

## Supporting information

Supporting information.

Supporting information.

Supporting information.

## Data Availability

The data used to support the findings of this study were provided within the article and the Supporting Information S1: Material 1 and Supporting Information S2: Material 2.

## References

[1] Fajt ML , Wenzel SE . Asthma phenotypes and the use of biologic medications in asthma and allergic disease: the next steps toward personalized care. J Allergy Clin Immunol. 2015;135(2):299‐310. 25662302 10.1016/j.jaci.2014.12.1871

[2] Manise M , Bakayoko B , Schleich F , Corhay JL , Louis R . IgE mediated sensitisation to aeroallergens in an asthmatic cohort: relationship with inflammatory phenotypes and disease severity. Int J Clin Pract. 2016;70(7):596‐605. 27352803 10.1111/ijcp.12837 PMC5094514

[3] Hammad H , Lambrecht BN . The basic immunology of asthma. Cell. 2021;184(6):1469‐1485. 33711259 10.1016/j.cell.2021.02.016

[4] Chang HS , Lee T‐H , Jun JA , et al. Neutrophilic inflammation in asthma: mechanisms and therapeutic considerations. Expert Rev Respir Med. 2017;11(1):29‐40. 27918221 10.1080/17476348.2017.1268919

[5] Louis R , Satia I , Ojanguren I , et al. European Respiratory Society Guidelines for the diagnosis of asthma in adults. Eur Respir J. 2022;60:2101585. 10.1183/13993003.01585-2021 35169025

[6] Demarche SF , Schleich FN , Henket MA , Paulus VA , Van Hees TJ , Louis RE . Effectiveness of inhaled corticosteroids in real life on clinical outcomes, sputum cells and systemic inflammation in asthmatics: a retrospective cohort study in a secondary care centre. BMJ Open. 2017;7(11):e018186. 10.1136/bmjopen-2017-018186 PMC5719334 29183929

[7] Kuruvilla ME , Lee FE‐H , Lee GB . Understanding asthma phenotypes, endotypes, and mechanisms of disease. Clin Rev Allergy Immunol. 2019;56(2):219‐233. 30206782 10.1007/s12016-018-8712-1 PMC6411459

[8] Oeser K , Maxeiner J , Symowski C , Stassen M , Voehringer D . T cells are the critical source of IL‐4/IL‐13 in a mouse model of allergic asthma. Allergy. 2015;70(11):1440‐1449. 26214396 10.1111/all.12705

[9] Russkamp D , Aguilar‐Pimentel A , Alessandrini F , et al. IL‐4 receptor α blockade prevents sensitization and alters acute and long‐lasting effects of allergen‐specific immunotherapy of murine allergic asthma. Allergy. 2019;74(8):1549‐1560. 30829405 10.1111/all.13759

[10] Xie Y , Abel PW , Casale TB , Tu Y . TH17 cells and corticosteroid insensitivity in severe asthma. J Allergy Clin Immunol. 2022;149(2):467‐479. 34953791 10.1016/j.jaci.2021.12.769 PMC8821175

[11] Yang X , Li H , Ma Q , Zhang Q , Wang C . Neutrophilic asthma is associated with increased airway bacterial burden and disordered community composition. BioMed Res Int. 2018;2018:9230234. 30105264 10.1155/2018/9230234 PMC6076954

[12] Hauk PJ , Krawiec M , Murphy J , et al. Neutrophilic airway inflammation and association with bacterial lipopolysaccharide in children with asthma and wheezing. Pediatr Pulmonol. 2008;43(9):916‐923. 18668688 10.1002/ppul.20880

[13] Carr TF . Treatment approaches for the patient with T2 low asthma. Ann Allergy Asthma Immunol. 2021;127(5):530‐535. 34688426 10.1016/j.anai.2021.05.027

[14] Camargo LdN , Righetti RF , Aristóteles LRdCRB Dos , et al. Effects of anti‐IL‐17 on inflammation, remodeling, and oxidative stress in an experimental model of asthma exacerbated by LPS. Front Immunol. 2017;8:1835. 29379497 10.3389/fimmu.2017.01835 PMC5760512

[15] Manni ML , Mandalapu S , McHugh KJ , Elloso MM , Dudas PL , Alcorn JF . Molecular mechanisms of airway hyperresponsiveness in a murine model of steroid‐resistant airway inflammation. J Immunol. 2016;196(3):963‐977. 26729801 10.4049/jimmunol.1501531 PMC4724491

[16] Ricciardolo FLM , Sprio AE , Baroso A , et al. Characterization of T2‐low and T2‐high asthma phenotypes in real‐life. Biomedicines. 2021;9(11):1684. 34829913 10.3390/biomedicines9111684 PMC8615363

[17] Nabe T . Steroid‐resistant asthma and neutrophils. Biol Pharm Bull. 2020;43(1):31‐35. 31902928 10.1248/bpb.b19-00095

[18] El‐Gammal A , Oliveria J‐P , Howie K , et al. Allergen‐induced changes in bone marrow and airway dendritic cells in subjects with asthma. Am J Respir Crit Care Med. 2016;194(2):169‐177. 26844926 10.1164/rccm.201508-1623OC

[19] Brassard J , Maheux C , Langlois A , et al. Lipopolysaccharide impacts murine CD103+ DC differentiation, altering the lung DC population balance. Eur J Immunol. 2019;49(4):638‐652. 30707446 10.1002/eji.201847910

[20] Plantinga M , Guilliams M , Vanheerswynghels M , et al. Conventional and monocyte‐derived CD11b(+) dendritic cells initiate and maintain T helper 2 cell‐mediated immunity to house dust mite allergen. Immunity. 2013;38(2):322‐335. 23352232 10.1016/j.immuni.2012.10.016

[21] Suzuki Y , Maazi H , Sankaranarayanan I , et al. Lack of autophagy induces steroid‐resistant airway inflammation. J Allergy Clin Immunol. 2016;137(5):1382‐1389.e9. 26589586 10.1016/j.jaci.2015.09.033 PMC4860134

[22] Nakano H , Free ME , Whitehead GS , et al. Pulmonary CD103(+) dendritic cells prime Th2 responses to inhaled allergens. Mucosal Immunol. 2012;5(1):53‐65. 22012243 10.1038/mi.2011.47 PMC3697034

[23] Masuda C , Miyasaka T , Kawakami K , et al. Sex‐based differences in CD103+ dendritic cells promote female‐predominant Th2 cytokine production during allergic asthma. Clin Exp Allergy. 2018;48(4):379‐393. 29288569 10.1111/cea.13081

[24] Norimoto A , Hirose K , Iwata A , et al. Dectin‐2 promotes house dust mite‐induced T helper type 2 and type 17 cell differentiation and allergic airway inflammation in mice. Am J Respir Cell Mol Biol. 2014;51(2):201‐209. 24588637 10.1165/rcmb.2013-0522OC

[25] Ito T , Hirose K , Norimoto A , et al. Dectin‐1 plays an important role in house dust mite‐induced allergic airway inflammation through the activation of CD11b+ dendritic cells. J Immunol. 2017;198(1):61‐70. 27852745 10.4049/jimmunol.1502393

[26] Manners S , Alam R , Schwartz DA , Gorska MM . A mouse model links asthma susceptibility to prenatal exposure to diesel exhaust. J Allergy Clin Immunol. 2014;134(1):63‐72.e7. 24365139 10.1016/j.jaci.2013.10.047 PMC4065237

[27] Decaesteker T , Vanhoffelen E , Trekels K , et al. Differential effects of intense exercise and pollution on the airways in a murine model. Part Fibre Toxicol. 2021;18(1):12. 33722268 10.1186/s12989-021-00401-6 PMC7962283

[28] Schlitzer A , McGovern N , Teo P , et al. IRF4 transcription factor‐dependent CD11b+ dendritic cells in human and mouse control mucosal IL‐17 cytokine responses. Immunity. 2013;38(5):970‐983. 23706669 10.1016/j.immuni.2013.04.011 PMC3666057

[29] Persson EK , Uronen‐Hansson H , Semmrich M , et al. IRF4 transcription‐factor‐dependent CD103(+)CD11b(+) dendritic cells drive mucosal T helper 17 cell differentiation. Immunity. 2013;38(5):958‐969. 23664832 10.1016/j.immuni.2013.03.009

[30] He YQ , Qiao YL , Xu S , et al. Allergen induces CD11c(+) dendritic cell autophagy to aggravate allergic rhinitis through promoting immune imbalance. Int Immunopharmacol. 2022;106:108611. 35158226 10.1016/j.intimp.2022.108611

[31] Monaci S , Aldinucci C , Rossi D , et al. Hypoxia shapes autophagy in LPS‐activated dendritic cells. Front Immunol. 2020;11:573646. 33329536 10.3389/fimmu.2020.573646 PMC7734254

[32] Caucheteux SM , Hu‐Li J , Mohammed RN , Ager A , Paul WE . Cytokine regulation of lung Th17 response to airway immunization using LPS adjuvant. Mucosal Immunol. 2017;10(2):361‐372. 27328989 10.1038/mi.2016.54 PMC5179326

[33] Pham DL , Kim SH , Losol P , et al. Association of autophagy related gene polymorphisms with neutrophilic airway inflammation in adult asthma. Korean J Intern Med. 2016;31(2):375‐385. 26701229 10.3904/kjim.2014.390 PMC4773719

[34] Ong OC , Hu K , Rong H , Lee RH , Fung BKK . Gene structure and chromosome localization of the G gamma c subunit of human cone G‐protein (GNGT2). Genomics. 1997;44(1):101‐109. 9286705 10.1006/geno.1997.4814

[35] Liu G‐M , Ji X , Lu T‐C , et al. Comprehensive multi‐omics analysis identified core molecular processes in esophageal cancer and revealed GNGT2 as a potential prognostic marker. World J Gastroenterol. 2019;25(48):6890‐6901. 31908393 10.3748/wjg.v25.i48.6890 PMC6938725

[36] Kaiko GE , Phipps S , Angkasekwinai P , Dong C , Foster PS . NK cell deficiency predisposes to viral‐induced Th2‐type allergic inflammation via epithelial‐derived IL‐25. J Immunol. 2010;185(8):4681‐4690. 20855881 10.4049/jimmunol.1001758

[37] Ito T , Wang Y‐H , Duramad O , et al. TSLP‐activated dendritic cells induce an inflammatory T helper type 2 cell response through OX40 ligand. J Exp Med. 2005;202(9):1213‐1223. 16275760 10.1084/jem.20051135 PMC2213234

[38] Besnard A‐G , Togbe D , Guillou N , Erard F , Quesniaux V , Ryffel B . IL‐33‐activated dendritic cells are critical for allergic airway inflammation. Eur J Immunol. 2011;41(6):1675‐1686. 21469105 10.1002/eji.201041033

[39] Lewkowich IP , Lajoie S , Clark JR , Herman NS , Sproles AA , Wills‐Karp M . Allergen uptake, activation, and IL‐23 production by pulmonary myeloid DCs drives airway hyperresponsiveness in asthma‐susceptible mice. PLoS One. 2008;3(12):e3879. 19060952 10.1371/journal.pone.0003879 PMC2586658

[40] Fear VS , Lai SP , Zosky GR , et al. A pathogenic role for the integrin CD103 in experimental allergic airways disease. Physiol Rep. 2016;4(21):e13021. 27905296 10.14814/phy2.13021 PMC5112499

[41] Beaty SR , Rose Jr CE , Sung SJ . Diverse and potent chemokine production by lung CD11bhigh dendritic cells in homeostasis and in allergic lung inflammation. J Immunol. 2007;178(3):1882‐1895. 17237439 10.4049/jimmunol.178.3.1882

[42] Sung SSJ , Fu SM , Rose Jr CE , Gaskin F , Ju ST , Beaty SR . A major lung CD103 (αE)‐β7 integrin‐positive epithelial dendritic cell population expressing langerin and tight junction proteins. J Immunol. 2006;176(4):2161‐2172. 16455972 10.4049/jimmunol.176.4.2161

[43] von Garnier C , Wikstrom ME , Zosky G , et al. Allergic airways disease develops after an increase in allergen capture and processing in the airway mucosa. J Immunol. 2007;179(9):5748‐5759. 17947647 10.4049/jimmunol.179.9.5748

[44] Koustas E , Trifylli EM , Sarantis P , et al. Exploiting autophagy‐dependent neoantigen presentation in tumor microenvironment. Genes. 2023;14(2):474. 36833401 10.3390/genes14020474 PMC9956312

[45] He Y‐Q , Qiao Y‐L , Xu S , et al. Allergen induces CD11c+ dendritic cell autophagy to aggravate allergic rhinitis through promoting immune imbalance. Int Immunopharmacol. 2022;106:108611. 35158226 10.1016/j.intimp.2022.108611

[46] Bakakos A , Sotiropoulou Z , Vontetsianos A , Zaneli S , Papaioannou A , Bakakos P . Epidemiology and immunopathogenesis of virus associated asthma exacerbations. J Asthma Allergy. 2023;16:1025‐1040. 37791040 10.2147/JAA.S277455 PMC10543746

[47] Reed M , Morris SH , Owczarczyk AB , Lukacs NW . Deficiency of autophagy protein Map1‐LC3b mediates IL‐17‐dependent lung pathology during respiratory viral infection via ER stress‐associated IL‐1. Mucosal Immunol. 2015;8(5):1118‐1130. 25669150 10.1038/mi.2015.3 PMC4532659

[48] de Lima Thomaz L , Peron G , Oliveira J , da Rosa LC , Thomé R , Verinaud L . The impact of metabolic reprogramming on dendritic cell function. Int Immunopharmacol. 2018;63:84‐93. 30075432 10.1016/j.intimp.2018.07.031

[49] Xie B , Zhao M , Song D , et al. Induction of autophagy and suppression of type I IFN secretion by CSFV. Autophagy. 2021;17(4):925‐947. 32160078 10.1080/15548627.2020.1739445 PMC8078712

[50] Pantazi I , Papafragkos I , Kolliniati O , Lapi I , Tsatsanis C , Vergadi E . Akt inhibition promotes autophagy and clearance of group B streptococcus from the alveolar epithelium. Pathogens. 2022;11(10):1134. 36297190 10.3390/pathogens11101134 PMC9611837

[51] Mihaylova MM , Shaw RJ . The AMPK signalling pathway coordinates cell growth, autophagy and metabolism. Nature Cell Biol. 2011;13(9):1016‐1023. 21892142 10.1038/ncb2329 PMC3249400

[52] Cabezudo S , Sanz‐Flores M , Caballero A , et al. Gαq activation modulates autophagy by promoting mTORC1 signaling. Nat Commun. 2021;12(1):4540. 34315875 10.1038/s41467-021-24811-4 PMC8316552

[53] Ghislat G , Lawrence T . Autophagy in dendritic cells. Cell Mol Immunol. 2018;15(11):944‐952. 29578531 10.1038/cmi.2018.2 PMC6207777

[54] Germic N , Frangez Z , Yousefi S , Simon HU . Regulation of the innate immune system by autophagy: monocytes, macrophages, dendritic cells and antigen presentation. Cell Death Differ. 2019;26(4):715‐727. 30737475 10.1038/s41418-019-0297-6 PMC6460400

[55] Li T , Zhang Y , Han D , et al. Involvement of IL‐17 in secondary brain injury after a traumatic brain injury in rats. NeuroMolecular Med. 2017;19(4):541‐554. 28916896 10.1007/s12017-017-8468-4

[56] Du B , Zhang Z , Li N . Madecassoside prevents Aβ(25‐35)‐induced inflammatory responses and autophagy in neuronal cells through the class III PI3K/Beclin‐1/Bcl‐2 pathway. Int Immunopharmacol. 2014;20(1):221‐228. 24631516 10.1016/j.intimp.2014.02.036

[57] Gu S , Tan J , Li Q , et al. Downregulation of LAPTM4B contributes to the impairment of the autophagic flux via unopposed activation of mTORC1 signaling during myocardial ischemia/reperfusion injury. Circ Res. 2020;127(7):e148‐e165. 32693673 10.1161/CIRCRESAHA.119.316388

[58] Campbell AP , Smrcka AV . Targeting G protein‐coupled receptor signalling by blocking G proteins. Nat Rev Drug Discov. 2018;17(11):789‐803. 30262890 10.1038/nrd.2018.135 PMC6409483

[59] Li J , Ge Y , Huang JX , Strømgaard K , Zhang X , Xiong XF . Heterotrimeric G proteins as therapeutic targets in drug discovery. J Med Chem. 2020;63(10):5013‐5030. 31841625 10.1021/acs.jmedchem.9b01452

[60] Momotani K , Somlyo AV . p63RhoGEF: a new switch for G(q)‐mediated activation of smooth muscle. Trends Cardiovascul Med. 2012;22(5):122‐127. 10.1016/j.tcm.2012.07.007 PMC3472095 22902181

[61] Johnson EN , Druey KM . Heterotrimeric G protein signaling: role in asthma and allergic inflammation. J Allergy Clin Immunol. 2002;109(4):592‐602. 11941304 10.1067/mai.2002.122636

[62] Matera MG , Page C , Rinaldi B . β2‐Adrenoceptor signalling bias in asthma and COPD and the potential impact on the comorbidities associated with these diseases. Curr Opin Pharmacol. 2018;40:142‐146. 29763833 10.1016/j.coph.2018.04.012

[63] Wendell SG , Fan H , Zhang C . G protein‐coupled receptors in asthma therapy: pharmacology and drug action. Pharmacol Rev. 2020;72(1):1‐49. 31767622 10.1124/pr.118.016899 PMC6878000

[64] Singh RK , Tandon R , Dastidar SG , Ray A . A review on leukotrienes and their receptors with reference to asthma. J Asthma. 2013;50(9):922‐931. 23859232 10.3109/02770903.2013.823447

[65] Majumdar R , Tavakoli Tameh A , Arya SB , Parent CA . Exosomes mediate LTB4 release during neutrophil chemotaxis. PLoS Biol. 2021;19(7):e3001271. 34232954 10.1371/journal.pbio.3001271 PMC8262914

[66] Lee J , Kim TH , Murray F , et al. Cyclic AMP concentrations in dendritic cells induce and regulate Th2 immunity and allergic asthma. Proc Natl Acad Sci. 2015;112(5):1529‐1534. 25605931 10.1073/pnas.1417972112 PMC4321256

[67] Lai W , Cai Y , Zhou J , et al. Deficiency of the G protein Gαq ameliorates experimental autoimmune encephalomyelitis with impaired DC‐derived IL‐6 production and Th17 differentiation. Cell Mol Immunol. 2017;14(6):557‐567. 28216651 10.1038/cmi.2016.65 PMC5518818

[68] Zhou J , Lai W , Yang W , et al. BLT1 in dendritic cells promotes Th1/Th17 differentiation and its deficiency ameliorates TNBS‐induced colitis. Cell Mol Immunol. 2018;15(12):1047‐1056. 29670278 10.1038/s41423-018-0030-2 PMC6269524

[69] Su X , Pang YT , Li W , Gumbart JC , Kelley J , Torres M . N‐terminal intrinsic disorder is an ancestral feature of Gγ subunits that influences the balance between different Gβγ signaling axes in yeast. J Biol Chem. 2023;299(8):104947. 37354971 10.1016/j.jbc.2023.104947 PMC10393545

[70] Dong Z , Ma Y , Zhou H , et al. Integrated genomics analysis highlights important SNPs and genes implicated in moderate‐to‐severe asthma based on GWAS and eQTL datasets. BMC Pulm Med. 2020;20(1):270. 33066754 10.1186/s12890-020-01303-7 PMC7568423

[71] Qin J , Wuniqiemu T , Wei Y , et al. Proteomics analysis reveals suppression of IL‐17 signaling pathways contributed to the therapeutic effects of Jia‐Wei Bu‐Shen‐Yi‐Qi formula in a murine asthma model. Phytomedicine. 2022;95:153803. 34785105 10.1016/j.phymed.2021.153803

