# Supplementary material for: Bioinformatics analysis of G protein subunit gamma transduction protein 2‐autophagy axis in CD11b+ dendritic cells as a potential regulator to skew airway neutrophilic inflammation in asthma endotypes
Source: Immun Inflamm Dis. 2024 Oct 17;12(10):e70038. doi: 10.1002/iid3.70038 (PMC11484477; doi:10.1002/iid3.70038)
Supplement: Supplementary file 3 — Supporting information. [file IID3-12-e70038-s003.pdf]

Table1. The Regulation of Th Cell Differentiation and Activation by Autophagic Balance in DCs

Autophagy-related Targets	Stimulus type	Autophagy	The role of autophagy in CD4+T cell differentiation and activation	References
ATG5	OVA	up	stimulation of DCs with OVA resulted in an augmented expression of autophagy-related proteins, such as ATG5 and LCII, thereby facilitating the differentiation and cytokine production of Th2	Int Immunopharmacol. 2022;106:108611.
ATG5	HDM	down	$Atg5^{-/-}$ DC augments HDM induced Th17 mediated neutrophilic airway inflammation through elevated IL-1 and IL-23 pathway	J Allergy Clin Immunol. 2016;137(5):1382-89.e9.
ATG5	Toxoplasma gondii	down	$Atg5^{-/-}$ DCs enhances IL-2 and IFN-γ production by Toxoplasma gondii-reactive CD4+ T cells	Microbes Infect. 2015;17(4):275-84.
ATG5	Neriifolin	down	Neriifolin inhibits ATG5-dependent phagocytosis, thereby restraining in situ reactivation and accumulation of CD4+ T cells during central nervous system inflammation in experimental autoimmune encephalomyelitis	Proc Natl Acad Sci U S A. 2017 ;114(52):E11228-237.

ATG7	LPS	down	The upregulation of miR-146a-5p in DCs following LPS stimulation inhibits ATG7, resulting in the promotion of Th17 cell differentiation and the inhibition of Treg cell differentiation	J Biochem Mol Toxicol 2022;36(10):e23151.
ATG16L	Bacteroides fragilis	down	The absence of Atg16L in DCs inhibits the induction and function of Tregs following stimulation with Bacteroides fragilis	Science. 2016;352(6289):1116-20
ATG16L	High-fat diet	down	Atg16L$^{-/-}$ DCs expands aortic Treg cells, restricts the accumulation of Th1 cells, and reduces the development of atherosclerosis in response to a high-fat diet.	Circ Res. 2019;125(11):1019-1034
beclin-1	Respiratory Syncytial Virus	down	Respiratory syncytial vrus-stimulated beclin-1$^{+/}$ DCs with impaired autophagy activity enhance Th2 cytokine release and suppress IL-17 and IFN-γ levels compared to co-culture with wild type DCs	J Immunol. 2013 ;191(5):2526-37.
beclin-1	Influenza A	down	Compared to wild type DCs, Influenza A-infected Beclin-1$^{+/-}$ DCs show decreased effectiveness in promoting the differentiation of Th1, Th2, and Th17 cells.	Immunology. 2016;148(1):56-69.

disabled homolog 2	LPS	down	LPS stimulation downregulates Disabled homolog 2 in DCs, leading to decreased phagocytosis and an enhanced Th1 and Th17 immune response	Front Immunol. 2019; 27;10:304.
GPCRs	Cannabinoids	up	Cannabinoids promote the generation of Tregs through autophagy mediated by GRCPs stimulation in DCs	Mucosal Immunol. 2022 ;15(1):96-108.
ICOS	Antigen	down	In antigen-induced arthritis, mice with autophagy-deficient DCs show increased synovial inflammation, as well as cartilage and bone erosion. This is accompanied by a correlation with Treg-to-Th17 conversion, which is achieved by inhibiting ICOS.	Cell Rep. 2019;28(1):21-29.e6.
MAPK	Propranolol	down	Propranolol induces the release of IL-23 to promote Th17 activation by inhibiting autophagy through MAPK activation.	Autophagy 2020;16(8):1380-95
Map-LC3	Respiratory Syncytial Virus	down	RSV-infected LC3b$^{-/-}$ DCs show a downregulation in autophagosome formation, leading to an increase in IL-17a secretion when co-cultured with purified CD4+ T cells, compared to wild type DCs	Mucosal Immunol. 2015;8(5):1118-30.
Map-LC3	HIV	up	Targeting HIV antigens to autophagosomes in dendritic cells using a LC3 fusion protein enhances and expands HIV-specific CD4(+) T cell responses in a more efficient manner	J Immunol. 2016 Jul 15;197(2):517-32

Map1-LC3/beclin-1	β-Glucan	up	β-Glucan promotes Th1 priming and Treg differentiation by autophagy activation in DCs	Med Microbiol Immunol. 2019;208(1):39-48
My88	Respiratory Syncytial Virus	up	Respiratory Syncytial Virus infection induces DCs activation, promoting interactions with T cells and resulting in elevated levels of Th1, Th2, and Th17 cytokines such as IFN-γ and IL-17 by MyD88 and TRIF mediated autophagy activation	J Immunol. 2011 Oct 15; 187(8): 3953–61.
Sirtuin 1	Respiratory Syncytial Virus	down	Inhibition of SIRT1 downregulates RSV-induced autophagy and promotes the differentiation of Th2 and Th17 cells	J Immunol. 2015;195(4):1637-46
STAT1	LPS	down	DCs harboring gain-of-function mutations in STAT1 impair autophagy, resulting in an inability to induce Tregs and Th17 cells, while promoting the generation of IFNγ-producing T cells	Clin Immunol. 2023;246:109174.
